# Sustaining fermentation in high-gravity ethanol production by feeding yeast to a temperature-profiled multifeed simultaneous saccharification and co-fermentation of wheat straw

**DOI:** 10.1186/s13068-017-0893-y

**Published:** 2017-09-12

**Authors:** Johan O. Westman, Ruifei Wang, Vera Novy, Carl Johan Franzén

**Affiliations:** 1 Division of Industrial Biotechnology, Department of Biology and Biological Engineering, Chalmers University of Technology, Gothenburg, Sweden; 2 Present Address: Chr. Hansen A/S, Bøge Allé 10-12, 2970 Hørsholm, Denmark; 3 Institute of Biotechnology and Biochemical Engineering, Graz University of Technology, Graz, Austria; 4 Present Address: Forest Products Biotechnology, Faculty of Forestry, University of British Columbia, Vancouver, BC V6T1Z4 Canada

**Keywords:** Multifeed simultaneous saccharification and co-fermentation (SSCF), High gravity, Yeast viability, Ethanol inhibition, Temperature effect, Combined stress, Flocculation, Demonstration scale, Wheat straw, Modeling

## Abstract

**Background:**

Considerable progress is being made in ethanol production from lignocellulosic feedstocks by fermentation, but negative effects of inhibitors on fermenting microorganisms are still challenging. Feeding preadapted cells has shown positive effects by sustaining fermentation in high-gravity simultaneous saccharification and co-fermentation (SSCF). Loss of cell viability has been reported in several SSCF studies on different substrates and seems to be the main reason for the declining ethanol production toward the end of the process. Here, we investigate how the combination of yeast preadaptation and feeding, cell flocculation, and temperature reduction improves the cell viability in SSCF of steam pretreated wheat straw.

**Results:**

More than 50% cell viability was lost during the first 24 h of high-gravity SSCF. No beneficial effects of adding selected nutrients were observed in shake flask SSCF. Ethanol concentrations greater than 50 g L^−1^ led to significant loss of viability and prevented further fermentation in SSCF. The benefits of feeding preadapted yeast cells were marginal at later stages of SSCF. Yeast flocculation did not improve the viability but simplified cell harvest and improved the feasibility of the cell feeding strategy in demo scale. Cultivation at 30 °C instead of 35 °C increased cell survival significantly on solid media containing ethanol and inhibitors. Similarly, in multifeed SSCF, cells maintained the viability and fermentation capacity when the temperature was reduced from 35 to 30 °C during the process, but hydrolysis yields were compromised. By combining the yeast feeding and temperature change, an ethanol concentration of 65 g L^−1^, equivalent to 70% of the theoretical yield, was obtained in multifeed SSCF on pretreated wheat straw. In demo scale, the process with flocculating yeast and temperature profile resulted in 5% (w/w) ethanol, equivalent to 53% of the theoretical yield.

**Conclusions:**

Multifeed SSCF was further developed by means of a flocculating yeast and a temperature-reduction profile. Ethanol toxicity is intensified in the presence of lignocellulosic inhibitors at temperatures that are beneficial to hydrolysis in high-gravity SSCF. The counteracting effects of temperature on cell viability and hydrolysis call for more tolerant microorganisms, enzyme systems with lower temperature optimum, or full optimization of the multifeed strategy with temperature profile.

**Electronic supplementary material:**

The online version of this article (doi:10.1186/s13068-017-0893-y) contains supplementary material, which is available to authorized users.

## Background

Ethanol produced from lignocellulosic materials by fermentation is one of the main alternatives for replacing fossil fuels in light-duty transportation. In recent years, the commercialization of lignocellulosic ethanol has gained momentum because of two factors: the improvement of enzymes, including reduced cost and the application of lytic polysaccharide monooxygenases in enzyme cocktails [1, 2], and the construction of several commercial-scale production plants [3, 4]. However, inhibitors formed during thermochemical pretreatment of lignocelluloses, which hinder the growth and fermentation ability of the microorganisms, present a major challenge [5]. Washing the pretreated slurry removes the inhibitors and leads to rapid fermentation, but also leads to loss of fermentable sugars and increased demands for wastewater treatment [6, 7].

A number of other strategies to reduce deleterious effects of inhibitors have been studied. For example, in situ detoxification has been demonstrated using sulfur oxyanions to sulfonate inhibitory compounds and render them less reactive [8], and laccase has been used to oxidize and polymerize free phenols [9]. The yeast *Saccharomyces cerevisiae* is one of the most-used fermenting microorganisms for its efficient glucose utilization and high stress tolerance in general. The robustness of yeast cells has been improved by genetic engineering for enhanced capacities in converting inhibitors and maintaining energy/redox balances [10–13]. Knowledge on the mechanisms governing inhibitor tolerance and cellular detoxification in yeasts and other microorganisms is thus of great importance [14–17]. Desirable traits in inhibitor resistance have also been obtained through mutagenesis, genome shuffling, and evolutionary engineering [18], where a detailed understanding of the mechanisms determining microbial resistance to individual or multiple stresses is not necessary [19]. Pre-exposure of cells to inhibitors during cultivation has been shown to effectively improve fermentation performance [20, 21]. The intrinsic detoxification capability of yeast cells has been exploited by using large amounts of inocula [22, 23], increasing the local cell density by encapsulation [24] or flocculation [25], and by continuous fermentation with cell retention [26]. Furthermore, proper implementation of rapidly sedimenting flocculating cells in bioprocesses gives the possibility to omit energy-intensive centrifugation steps [27].

Fed-batch simultaneous saccharification and co-fermentation (SSCF) can minimize the effects of inhibitors on the process, as the rate at which the inhibitors are introduced can be controlled [10]. In fed-batch SSCF, pretreated raw material is fed to the fermenter where it is continuously hydrolyzed and the released sugars are simultaneously fermented into ethanol. Substrate feeding is a practical solution in processes with high solid substrate loadings, also called high-gravity fermentation [28], and promotes xylose and glucose co-consumption [29]. In contrast to other processes for handling high substrate loadings, such as separate hydrolysis and co-fermentation and combined pre-hydrolysis and SSCF, large amounts of glucose are not accumulated in fed-batch SSCF due to the continuous removal of sugars by fermentation. A high xylose-to-glucose ratio can thus be maintained throughout the process, facilitating xylose utilization by recombinant xylose-consuming yeasts by relieving the competitive inhibition by glucose on the xylose uptake via the common glucose/xylose transporters [30]. Further development of fed-batch SSCF has been reported toward multifeed SSCF, in which feedings of substrate, enzymes and cells into the SSCF reactor are coordinated in order to balance the main reactions in the process, i.e., to maintain high and balanced rates of both hydrolysis and fermentation [31, 32].

Loss of cell viability has been reported in several studies on multifeed, high-gravity SSCF of different substrates [7, 31–33]. The lack of viable cells seems to be the main reason for the declining ethanol production toward the end of the process, which may lead to incomplete utilization of the available sugars and low overall ethanol yield. Possible reasons for the reduction in viability could be lignocellulose-derived inhibitors [34], lack of nutrients/nitrogen sources [7, 35], lack of unsaturated fatty acids and ergosterol for anaerobic conditions [36], problems associated with high-gravity process or viscous media, e.g., limited mass transfer, high osmolality, and slow regulation of pH and temperature [37], and toxicity of the ethanol produced [38].

In this study, we investigate the reasons for the decline in viable cell concentrations in high-gravity SSCF of pretreated wheat straw. It has been reported that yeast flocculation leads to improved tolerance to lignocellulose-derived inhibitors [25] and ethanol [39]. The effects of yeast flocculation, to improve cell robustness in general, and also to improve the feasibility of cell feeding during multifeed SSCF, were investigated. The knowledge obtained from these developments of the multifeed SSCF process will be valuable in the further development of flexible and robust lignocellulose-based processes and cell factories, with the objectives to increase the final product concentration and promote complete conversion of the carbohydrates in the lignocellulosic raw material.

## Methods

### Strains

The metabolically and evolutionarily engineered xylose-fermenting *S. cerevisiae* strains KE6-12.A (originating from the hybrid diploid wine yeast USM21 via TMB3400) [Albers et al. unpublished, 40, 41, 42] and IBB10B05 (originating from CEN.PK 113-5D) [43, 44] were used in this study. Both strains were transformed with a gene cassette containing the chimeric flocculation gene *FLOw* (GenBank accession number KT264162) [45]. The cassette was amplified from the genomic DNA of CEN.PK Flow (originating from *S. cerevisiae* CEN.PK 113-7D (MATa, MAL2-8C, SUC2) [46] with the forward primer 5´-CAGAAAGGGTTCGCAAGTC-3´ and reverse primer 5´-GGCGTATTTCTACTCCAGCATTC-3´ [45]. The resulting PCR product, with flanking regions homologous to the *HO* locus, was used for homologous recombination in the two parental strains mentioned above using the lithium acetate based transformation method [47]. Transformants were selected on yeast extract, peptone, dextrose (YPD) plates containing 10 g L^−1^ yeast extract (Difco™, BD Biosciences, San Jose, CA), 20 g L^−1^ peptone (Bacto™, BD Biosciences), 20 g L^−1^ glucose (d-glucose monohydrate, Merck KGaA, Darmstadt, Germany), 20 g L^−1^ agar (Merck KGaA) and 200 µg/mL G418 (Sigma-Aldrich, Steinheim, Germany). Correct integration into the *HO* locus (which in itself should not affect cell growth [48]) and the size of the gene were confirmed by PCR using the forward primer 5´-ATGATATCCAGTTCGAGTTTATCATTATC-3´ and the reverse primer 5´-CAAATCAGTGCCGGTAACG-3´. The resulting flocculating strains were named KE-Flow and B-Flow.

### Raw materials

Pretreated wheat straw and molasses used in this study were provided by SP Biorefinery Demo Plant (Örnsköldsvik, Sweden). The wheat straw was steam pretreated in a one-step vertical continuous reactor with the addition of 0.2% (w/w) H_2_SO_4_, at pH 2.1–2.2 and 187–188 °C for 6–7 min, by using 11.5 bar steam. After pretreatment, the biomass slurry was separated into a solid fraction and a liquid fraction (pretreatment liquor) using a filter press. The water-insoluble solids (WIS) content in the solid fraction was determined by washing a weighed amount of the moist solid fraction with excess deionized water (the wash was repeated until the glucose remaining in the wash liquid was less than 0.05 g L^−1^), before drying in an oven at 105 °C for 24 h and weighing of the residual dried material [49]. The solid fraction, in its moist state, was used for hydrolysis and SSCF experiments. The pH of the pretreament liquor was adjusted to 5 using 50% (w/w) NaOH, and it was then filtered by vacuum filtration through sterile disposable bottle top filters with 0.2 µm PES membrane (catalog number 597–4520, ThermoFisher Scientific, Waltham, MA, USA). The filtered liquor was used in yeast propagation, spotting assays, and the SSCF experiments. The sugar composition in the solid fraction was determined using the National Renewable Energy Laboratory (NREL) analytical procedure [50].

Two batches of pretreated wheat straw, as described previously [32], were used for experiments in shake flasks and 3.6 L Labfors (INFORS HT, Switzerland) bioreactors in this study, and a third batch was used in experiments at demonstration scale and in a 30 L Techfors (INFORS HT, Switzerland) laboratory bioreactor. Material must be produced before each demonstration-scale experiment as the quantity required was too large for long-term storage. The three batches were denoted M1, M2, and M3, and their solid and liquid phases had different compositions (Table 1).

**Table 1:** Compositions of solid and liquid fractions for three batches of pretreated wheat straw

Solid fraction (% WIS)				Liquid fraction (g L^−1^)			
	M1^a^	M2^a^	M3		M1^a^	M2	M3
Glucan	47.7 ± 3.5	42.4 ± 1.3	36.2 ± 5.0	Glucose	6.8	2.6 ± 0.1^b^	4.5 ± 0.2^b^
Xylan	2.3 ± 0.4	2.6 ± 0.1	4.3 ± 0.1	Xylose	12.8	22.8 ± 1.1^b^	32.7 ± 1.0^b^
Mannan	0.2 ± 0.1	0.2 ± 0.1	0.2 ± 0.08	Mannose	0.4	0.5 ± 0.1^b^	1.1 ± 0.1^b^
Galactan	0.04 ± 0.01	0	0.01 ± 0.00	Galactose	1.0	1.0 ± 0.2^b^	1.9 ± 0.1^b^
Arabinan	0.1 ± 0.01	0.08 ± 0.01	0.2 ± 0.00	Arabinose	2.0	2.8 ± 0.03^b^	4.2 ± 0.1^b^
Lignin		41.7	35.6	Acetic acid	3.8	3.2	3.7 ± 0.05
				Furfural	4.0	0.8	1.5 ± 0.04
Total		87.0	76.5	HMF	1.4	0.4	0.3 ± 0.00

All data are average of at least two separate measurements. Data shown are means ± range. M1, M2 and M3 stand for material 1, 2 and 3 received from the SP biorefinery demo plant. The ranges for acetic acid, furfural, and HMF are within 5% of the average

^a^Data from [32]

^b^Monomeric and oligomeric sugars

### Aerobic batch and fed-batch seed cultivation

The optimal composition of growth media was evaluated separately for batch and fed-batch phases; the batch medium in shake flasks at 35 °C and 200 rpm, and the fed-batch medium in bioreactors. The batch medium contained molasses as the main carbon source supply, and pretreatment liquor to adapt the cells to the toxic environment of SSCF. The medium also contained 7.5 g L^−1^ (NH_4_)_2_SO_4_, 3.5 g L^−1^ KH_2_PO_4_, 0.7 g L^−1^ MgSO_4_·7H_2_O, 2 mL L^−1^ trace metal (TM) solution and 1 mL L^−1^ vitamin solution. The TM and vitamin solutions were prepared as described previously [51]. Medium compositions were investigated at combinations of 5, 7.5, and 10% (v/v) molasses, and 20, 25, and 30% pretreatment liquor. The combination that gave the best compromise between high biomass and low ethanol yield during the 24-h aerobic cultivation, showing complete utilization of the sugars, was selected for batch propagation. The fed-batch medium contained salts as in the batch medium, but no addition of vitamin or TM solutions. The fed-batch phase was started after 24 h of aerobic batch culture. Molasses contents of 0, 2.5, and 5% were investigated in the feed medium, together with 80% pretreatment liquor. The minimal molasses content that produced enough cells for SSCF was selected for feed medium.

Cells from a glycerol stock kept at −80 °C were streaked out on a YPD plate and incubated at 30 °C for approximately 2 days. The liquid inoculum culture was inoculated with a single colony from the plate into 50–100 mL batch medium in 250–500 mL Erlenmeyer flasks and was incubated at 30 °C and a shaker speed of 200 rpm for approximately 24 h, after which the cells were harvested by centrifugation at 3000*g* for 3 min at 4 °C. Flocculating cells were deflocculated by resuspension in 50 mM EDTA followed by washing with 0.9% NaCl solution and final resuspension in 0.9% NaCl. The batch phase of the cell propagation was inoculated to an optical density (OD_600_) of 0.5 by addition of the required amount of cell suspension.

The yeast cells used in SSCF were propagated in aerobic batch (0.5 L working volume in 3.6 L Labfors bioreactor, INFORS HT, Switzerland), followed by fed-batch culture, at a dilution rate of 0.05 h^−1^, sparged with air, with aeration set to 1 vvm, 800 rpm stirring, a temperature of 35 °C and pH set to 5.0 by the addition of 3 M NaOH. The working volume was increased to about 2 L in the fed-batch phase and decreased to 0.5 L by harvesting part of the culture.

At every cell addition to the SSCF reactors, cells were harvested from the propagation reactor. For non-flocculating cells, OD_600_ of the culture was measured, and a separately determined correlation between the OD and the dry cell mass concentration was used to determine how much culture should be harvested. Centrifugation was performed at 3000*g* for 3 min at 4 °C, and the cell pellet was re-suspended and added to the SSCF reactors. Flocculating cells were allowed to sediment in a standing bottle. The supernatant was poured off and a concentrated cell suspension was obtained. An aliquot of the cell suspension (about 10 mL) was centrifuged at 3000*g* for 3 min at 4 °C. A separately determined correlation between the pellet volume and the dry cell weight [cell dry weight (g) = pellet volume (mL)*0.27; *R* ^2^ of the linear regression was 0.99] was used to determine the cell concentration in the suspension, and thus how much suspension should be added to the SSCF reactors.

### SSCF in shake flasks

The Cellic Ctec 2 enzyme preparation (Novozymes, Denmark) was used in all SSCF experiments. The cellulase activity of the enzyme preparation was 150 ± 7 FPU mL^−1^, measured according to the NREL protocol TP-510-42,628 [52]. Shake flask batch SSCF was carried out with working weight of 100 g in 250 mL baffled Erlenmeyer flasks as described previously [32]. Briefly, the solid fraction of pretreated wheat straw was added to the WIS content that is specified under the corresponding figures. The pH was initially adjusted to 5.0. The medium was supplemented with 0.5 g kg^−1^ (NH_4_)_2_HPO_4_. When using 20% WIS, the mixture of solids and enzymes, at 10 FPU g WIS^−1^, was preincubated at 50 °C for 2 h prior to cell inoculation, to allow partial liquefaction and mixing of the medium. SSCF was initiated by the addition of yeast cells at a cell loading of 0.02 g cell g WIS^−1^, after reducing the temperature to 35 °C. This cell loading has previously been shown to be sufficient for efficient fermentation in shake flask SSCF on 20% WIS [32]. The flasks were shaken at 180 rpm with no pH control during fermentation. Samples were taken every 24 h for cell viability and HPLC analysis.

### SSCF in bioreactors

Multifeed SSCF was performed at working weight around 1 kg in 3.6 L Labfors with one pitched blade impeller and two Rushton impellers, and in 30 L Techfors bioreactors with two Rushton impellers (INFORS HT, Switzerland). The solid fraction of pretreated wheat straw and yeast cells from a separate propagation reactor were added pulse-wise during the process. All the enzymes were added at the beginning of the process to a concentration of 10 FPU g^−1^ overall WIS, as this has previously been shown to be a suitable process option for wheat straw [32]. The medium was supplemented with 0.5 g (NH_4_)_2_HPO_4_ and 125 µL Vitahop (a hop-derived fermentation enhancer, BetaTech GmbH, Schwabach, Germany) per total kg of working weight, added to reduce the risk of contamination. The reactors were operated without gas sparging.

All the experiments were started at an initial WIS of 7%. Solids were added according to predetermined hydrolysis kinetics [32]. Detailed feed profiles can be found in Additional file 1: Table S1. In the faster feeding profile, feeding was initiated after hydrolysis of 50%, rather than 60%, of the cellulose, according to model predictions, and material was fed to temporarily reach 14% (w/w) calculated apparent WIS in the reactor, rather than 13% (w/w). Samples were collected for the analysis of sugars, fermentation products, and residual inhibitors. Multifeed SSCF in bioreactors was carried out at 35 °C, however, in some experiments the temperature was reduced to 30 °C after 24 or 48 h. The pH was controlled at 5.0 by the addition of 3 M NaOH, and the agitation rate was 400 rpm.

### Determination of cell concentration and viability

The cell concentration during aerobic propagation was determined by cell dry weight (CDW) and OD_600_. Upon harvest, the flocculating yeast strains KE-Flow and B-Flow were deflocculated with 50 mM EDTA and washed with 0.9% (w/v) NaCl solution before measurement. The CDW was measured by first filtering the cell solution through a preweighed 0.45 µm filter (Sartorius, Göttingen, Germany), drying for 15 min at 150 W in a microwave oven, and cooling in a desiccator before weighing. The viable cell concentration was determined as colony forming units (CFU) during both cell propagation and SSCF. CFU were determined by plating 0.1 mL of sample, after serial dilution with 0.9% (w/v) NaCl, on YPD plates. The plates were incubated for approximately 2 days at 30 °C before colony counting. The total cell concentration was determined by counting cells under a light microscope (Leica DM 2000) in a Neubauer improved hemocytometer (Assistent, Glaswarenfabrik Karl Hecht, Germany).

### Spotting assays

Concentrated (2.5×) YPD agar medium was mixed with filtered pretreatment liquor, ethanol, or both to prepare plates of 1 × YPD agar medium containing in addition to YPD agar, 50% (v/v) liquor, or 50 g L^−1^ ethanol or both. Upon harvest, the KE-Flow and B-Flow cells were treated with 50 mM EDTA, resulting in complete deflocculation, and washed with 0.9% (w/v) NaCl solution. The deflocculated cells were re-suspended to an OD_600_ of approximately 0.05. Twofold serial dilutions were made from the cell suspension, and 50 μL per dilution was spotted onto the plates. The plates were incubated at 30 or 35 °C for 2 days, after which they were examined and photographed using a Gel Doc scanner (Bio-Rad, Hercules, CA).

### Analysis of sugars, fermentation products, and inhibitors

Fermentation samples were filtered through 0.2-µm nylon filters (VWR, Radnor, PA) prior to storage at −20 °C until analysis. The concentrations of the sugars (glucose, xylose, arabinose, galactose, and mannose) were analyzed using high-performance anion exchange chromatography on an ICS 3000 system (Dionex, Thermo Scientific, Sunnyvale, CA) with guard and analytical CarboPac (PA1) columns (Thermo Scientific) maintained at 30 °C, and by electrochemical detection. Milli-Q water was used for sample elution at a flow rate of 1 mL min^−1^, and 300 mM NaOH was added post-column at a flow rate of 0.5 mL min^−1^ before the detector. The column was regenerated between sample injections using a mixed eluent consisting of (by volume) 20% Milli-Q water, 40% 300 mM NaOH, and 40% 100 mM NaOH + 85 mM sodium acetate, followed by equilibration with Milli-Q water. The fermentation products (glycerol, xylitol, and ethanol) and inhibitors [acetic acid, furfural, and 5-(hydroxymethyl)furfural] were analyzed using high-performance liquid chromatography on a Dionex Ultimate 3000 system (Dionex, Thermo Scientific) equipped with a Phenomenex Rezex ROA column (Phenomenex, Torrance, CA) and a refractive index (RI) detector (Shodex, SHOWA DENKO K.K., Tokyo, Japan). Samples were eluted at 80 °C using 5 mM H_2_SO_4_ at flow rate of 0.8 mL min^−1^. The concentrations were calculated from calibration curves for standard solutions.

## Results and discussion

### Ethanol is a major inhibitor in high-gravity lignocellulosic ethanol production

**Fig. 1:**
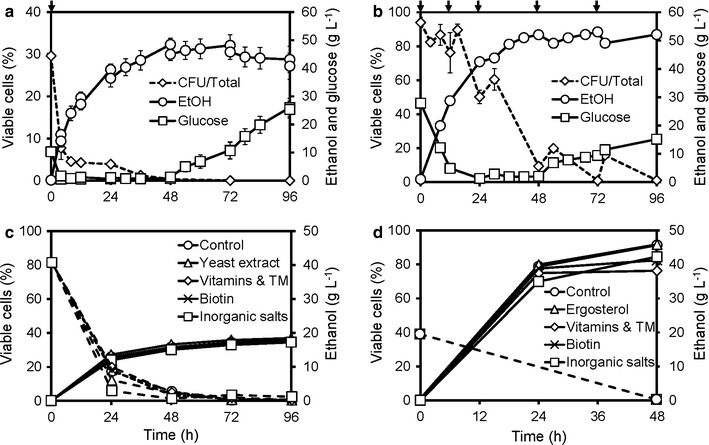
Decrease of cell viability during multifeed SSCF and limited effects of nutrient supplementation. Cell viability (% of CFU/total cell counts) and concentrations of glucose and ethanol during **a** laboratory-scale SSCF of material M1 with all the yeast added initially; and **b** demonstration-scale SSCF with feeding of yeast and substrate material M2. Ethanol concentration and cell viability during **c** 7% WIS and **d** 20% WIS (with 2-h pre-hydrolysis) shake flask SSCF of material M1 with additions of indicated nutrients. *S. cerevisiae* KE6-12.A was used, and the temperature was 35 °C in all cases. *Arrows* in (**a**–**b**) indicate the loading of yeast cells. *TM* trace metals. *Error bars* show the results of duplicate experiments. *Error bars* for ethanol concentration in (**c**–**d**) are smaller than the *symbols*. The measured cell viability in (**d**) was 0 at 48 h for all cases. Panels **a** and **b** are adapted from [32]

In the fermentation of pretreated wheat straw by *S. cerevisiae* KE6-12.A in the multifeed SSCF process, the concentration of viable cells (measured by CFU) decreased to almost zero during the process, regardless of whether more or less toxic pretreated wheat straw were used, and regardless of the scales of the process (Fig. 1a, b). Similar observations have been reported with various raw materials (wheat straw, birch, and spruce) [31–33]. These results indicated that the fermentation conditions in these lignocellulosic media were not suitable for sustaining cell viability. Cell feeding, instead of adding all the cells at the beginning of fermentation, allowed for a more effective use of the same total amount of cells, and the fermentation capacity was maintained for a longer period. However, cell feeding was not sufficient to stop the decline in the viable cell population or to ensure complete fermentation of the released sugars at the end of the process (Fig. 1b).

To investigate whether the observed decrease in viability during SSCF was due to some nutrient limitation, yeast extract (2 g kg^−1^), vitamin solution (1 mL kg^−1^), trace metal (TM) solution (2 mL kg^−1^) [51], biotin (1 mg kg^−1^), or inorganic salts [7.5 g kg^−1^ (NH_4_)_2_SO_4_, 3.5 g kg^−1^ KH_2_PO_4_ and 0.7 g kg^−1^ MgSO_4_·7H_2_O] were added to shake flask SSCF at a low content of water insoluble solids (WIS, 7% w/w). None of them led to significantly improved ethanol production or an increase in cell viability (Fig. 1c) The addition of nutrients was further tested in 20% WIS shake flask SSCF, in which the inhibitor concentrations were much higher. The same amounts of vitamin and TM solutions, biotin, and inorganic salts as above were used. In addition, the anaerobic growth factor ergosterol (10 mg kg^−1^) and Tween 80 (420 mg kg^−1^) were added, but no improvements in ethanol production were observed, and the cell viability decreased to zero within 48 h in all cases (Fig. 1d). The medium composition in control experiments was the same as that in multifeed SSCF. It was therefore concluded that the absence of viable cells at later stages of multifeed SSCF was not due to nutrient limitation.

Other factors causing stress during multifeed SSCF are the inhibitors present in the raw material and the ethanol produced. However, inhibitor concentrations do not increase to high levels in a fed-batch process due to dilution effects and continuous conversion by the yeast. In fact, a decrease in aromatic aldehyde concentrations and an almost constant acetic acid concentration were observed (data not shown). The only potentially toxic compound that increased significantly in concentration over time was the fermentation product, ethanol. Ethanol inhibition has so far not been seen as a major problem in lignocellulosic ethanol production, and has only been mentioned as a potential issue in high-gravity fermentation [37]. It is only recently that the ethanol titers reported from lignocellulosic feedstocks have exceeded the benchmark of 4% (w/w). These titers are still far below those achieved with sucrose- and starch-based substrates, of 8–12% (v/v) [53, 54], and lower than the levels generally considered to be lethal to the yeast *S. cerevisiae*. For example, the wild-type lab strain of BY4741 has been cultured in the presence of 10 and 12.5% (v/v) ethanol for 30 h, with remaining viabilities of 80 and 30%, respectively [55].

**Fig. 2:**
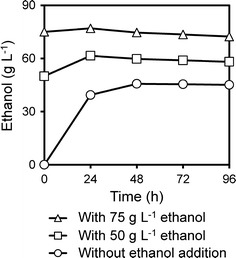
Ethanol inhibition in high-gravity SSCF with *S. cerevisiae* KE6-12.A. Shake flask SSCF of 20% (w/w) WIS of steam-pretreated wheat straw (material M1) with 2-h pre-hydrolysis, 10 FPU (g WIS)^−1^ enzyme dosage, at 35 °C. The addition of approximately 75 or 50 g L^−1^ ethanol severely inhibited ethanol production. Results shown are average values of duplicate experiments, and the relative difference between duplicate experiments was lower than 5%

To investigate the significance of inhibition by ethanol, different levels of ethanol were added to 20% (w/w) WIS shake flask SSCF experiments. Without ethanol addition, approximately 40 g L^−1^ ethanol was produced within the first 24 h (i.e., an average fermentation rate of 1.6 g L^−1^ h^−1^), after which the fermentation rate decreased to 0.1 g L^−1^ h^−1^ between 24 and 48 h. When approximately 50 g L^−1^ ethanol was added, some ethanol production was evident during the first 24 h (~0.5 g L^−1^ h^−1^), after which production ceased and the ethanol concentration decreased, probably due to evaporation (−0.04 g L^−1^ h^−1^ from 24 to 48 h). The amount of ethanol produced was only 25% of that produced in the control case. When 75 g L^−1^ ethanol was added at the start of fermentation, virtually no ethanol production was seen in the shake flasks (0.08 g L^−1^ h^−1^ from 0 to 24 h) (Fig. 2). These results clearly show that at concentrations of about 50 g L^−1^ and above, ethanol is already a major inhibitor of fermentation in SSCF with 20% WIS of pretreated wheat straw. A concentration range of 40–60 g L^−1^ of ethanol has been observed to be a turning point where drastic reduction in growth rate and prolongation of the lag-phase occur for several laboratory and industrial *S. cerevisiae* strains cultivated in the medium of spruce hydrolysate [56].

To investigate whether ethanol also inhibits the hydrolytic enzymes, enzymatic hydrolysis was carried out in shake flasks on 20% WIS with the addition of 75 g L^−1^ ethanol. The results showed a 12% decrease in glucose release (Additional file 1: Figure S1). Therefore, inhibition of the enzymes by ethanol was not the main reason for the observed decreased ethanol production in the SSCF experiments with ethanol supplementations.

From these results, it is clear that at concentrations of 40–50 g L^−1^, ethanol became inhibitory to *S. cerevisiae* KE6-12.A in SSCF at 20% WIS, with 0.02 g DW (g WIS)^−1^ inoculation. Our results indicate that in high-gravity lignocellulosic ethanol production, ethanol inhibition has to be alleviated so that further increases in ethanol titer would be possible.

### Flocculation simplifies cell feeding in the multifeed SSCF process

It has been shown that when cells agglomerate and form dense flocs, their tolerance to ethanol [39] and to furan aldehydes [25] is improved. Thus, flocculation could be a promising strategy to enhance the performance of *S. cerevisiae* in high-gravity, lignocellulosic ethanol production, especially to increase the ethanol titer. Furthermore, flocculating cells would rapidly sediment at the bottom of the reactor making cell recovery and processing easier.

**Fig. 3:**
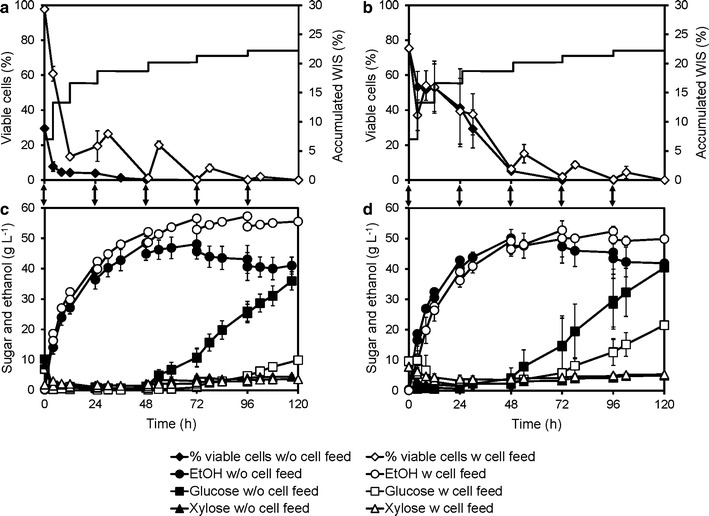
Multifeed SSCF with the non-flocculating strain KE6-12.A and the flocculating strain KE-Flow. Accumulated WIS concent and percent viability (**a**, **b**) and concentrations of ethanol, glucose, and xylose (**c**, **d**) using: (**a**, **c**) KE6-12.A; and (**b**, **d**) KE-Flow in multifeed SSCF at 22% (w/w) overall WIS of material M1, at 35 °C. Cells were added initially (w/o cell feed, *filled symbols*) or fed during the process (w cell feed, *open symbols*). Feeding of cells is indicated by the *arrows*. The detailed feeding profile has been described elsewhere [32]. Values are averages from duplicate experiments, and *error bars* show the results from the individual experiments

The flocculating strain KE-Flow formed dense cell aggregates approximately 3–4 mm in size when cultivated in shake flasks with liquid medium. However, no improvement was observed with the KE-Flow strain compared to that with the KE6-12.A strain in shake flask SSCF (Additional file 1: Figure S2), or multifeed SSCF in bioreactors, regardless of whether all the yeast was added initially or at different times (Fig. 3). Fermentation was so slow in the later stages of SSCF that the released glucose accumulated in the reactor, and the viability decreased despite the flocculation. This suggests that dense cell flocs were not successfully formed in the SSCF reactor [25]. The SSCF medium contained a high amount of WIS, i.e., undissolved particles. These particles, together with the mixing in the reactor, probably disrupted larger flocs. These results also suggest that flocculation might not protect cells from ethanol inhibition under the conditions used in SSCF.

**Fig. 4:**
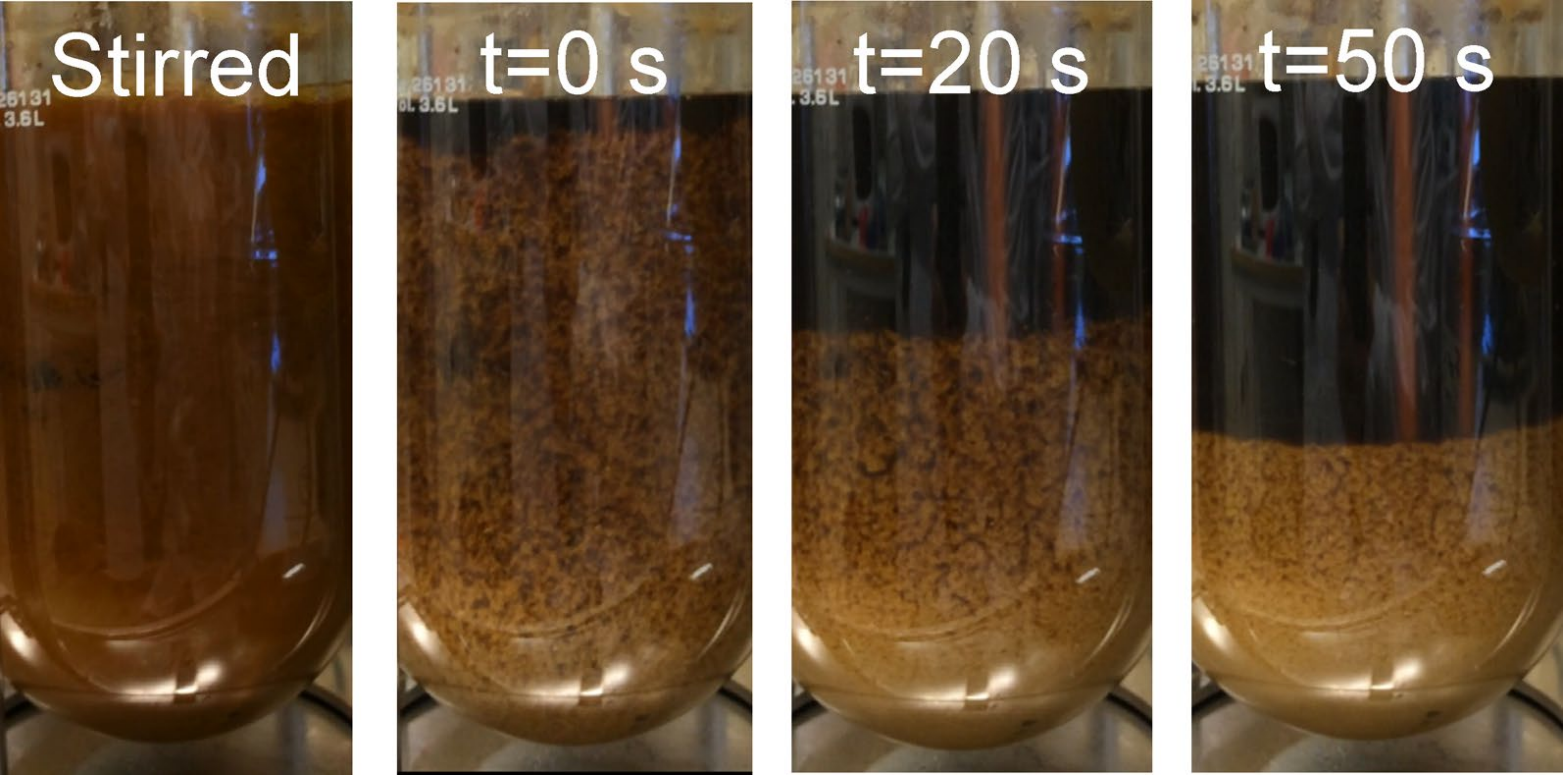
Sedimentation of KE-Flow cells in propagation reactor. At *t* = 0 s, stirring and aeration were completely stopped. Pretreatment liquor from material M2 was used in this case

Although no improvement in performance was observed in SSCF when using flocculating cells, rapid sedimentation of the KE-Flow cells was observed in the propagation reactor (Fig. 4). The strong flocculation is typical for the Flo1 phenotype [57], which is illustrated by these mutants. The strain may thus be attractive from a process point of view. Using flocculating cells may simplify the harvesting and feeding of cells to the SSCF reactor.

### High temperature exacerbates inhibition by ethanol and pretreatment liquor

As discussed above, inhibition by ethanol in high-gravity SSCF was greater than that expected for *S. cerevisiae*. It is unlikely that ethanol at a concentration of 40–50 g L^−1^ was solely responsible. Rather, the inhibition was due to the combined effect of ethanol, lignocellulose-derived inhibitors, and stressful SSCF conditions. In the SSCF or, generally, simultaneous saccharification and fermentation (SSF) setups, the temperature is a compromise between the optimal temperatures for the hydrolytic enzymes (45–50 °C for Cellic CTec2, Novozymes) and the fermenting microorganism (around 30 °C for *S. cerevisiae*) [58]. This may cause additional stress on the cells in the multifeed SSCF. Xiros and Olsson showed that using preadapted yeast cells and yeast extract improved the ethanol yield about 30 times at 30 °C in SSF of spruce at 20% WIS, but at higher temperatures, the effectivity of this strategy decreased [7]. These findings led us to hypothesize that the ethanol produced, in combination with the inhibitors and the relatively high temperature for the yeast (35 °C) caused the decrease in viability and fermentation capacity.

**Fig. 5:**
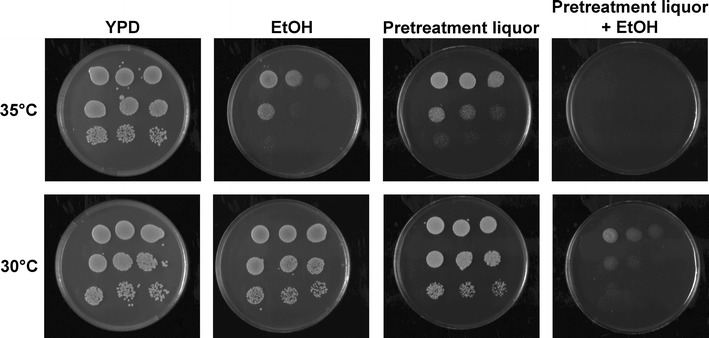
Spotting assay of the KE-Flow cells. YPD agar plates were supplemented with 50 g L^−1^ ethanol, or 50% (v/v) pretreatment liquor (from material M2), or both. The cells were harvested after 24 h of fed-batch propagation. The plates were incubated at 35 or 30 °C for 48 h. Duplicate cell dilution and plating showed similar results

To investigate the effects of combined stresses on yeast growth, spotting assays were performed at 35 and 30 °C. The results clearly showed that incubation at the higher temperature strongly increased the inhibitory effects of both ethanol and the pretreatment liquor (Fig. 5). With the combination of ethanol and pretreatment liquor, almost no cells grew at 35 °C, showing a strong additive, possibly synergistic, effect of the three factors resembling conditions in high-gravity SSCF. This is probably the reason why no fermentation was observed when a high concentration of ethanol was added to SSCF (Fig. 2), and why further fermentation was difficult with ethanol concentrations greater than 50 g L^−1^, in spite of adding fresh cells during the process.

Similar results were observed in spotting assays with cells obtained at different time points during the fed-batch cell propagation (data not shown). Ethanol and elevated temperature could affect both the fluidity and structure of the cell membrane [59, 60], making the cells more permeable [61] and thus vulnerable when toxic compounds are present. Similar results were also observed when the xylose-fermenting, evolutionary engineered *S. cerevisiae* strain IBB10B05 [43] was used (Additional file 1: Figure S3), suggesting that the combined inhibition due to ethanol, inhibitors, and temperature is not strain specific.

The YPD plates were incubated aerobically, indicating that the inability of the yeast to survive the combined stress was not due to a lack of sterols or oleic acid, since *S. cerevisiae* can synthesize these under aerobic conditions. This is consistent with the results of the ergosterol-supplemented batch SSCF experiments (Fig. 1d), in which the addition of sterols and oleic acid (Tween 80) did not improve the ethanol production.

### Reducing the temperature during SSCF improves cell viability

In order to achieve sustained fermentation and complete sugar utilization, the inhibition caused by ethanol, inhibitors, and the process temperature must be alleviated. Decreasing the temperature during SSCF could lead to improved cell viability and thus more complete fermentation. However, the risk of less complete hydrolysis has to be considered.

**Fig. 6:**
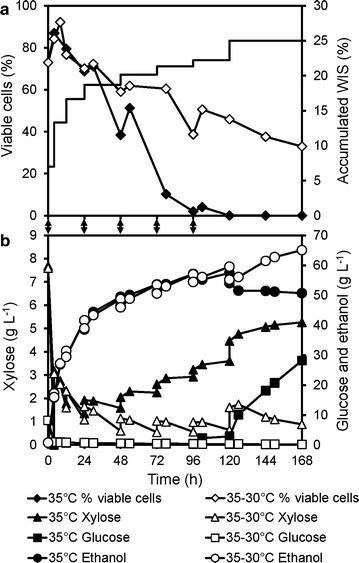
Multifeed SSCF at constant temperature or with temperature reduction. **a** Cell viability (% of CFU/total cell counts) and accumulated WIS content (indicating substrate feeding); and **b** concentration of glucose, xylose, and ethanol in 25% (w/w) WIS multifeed SSCF of material M2, with the KE-Flow strain, at a constant temperature of 35 °C, or at 35 °C for 24 h, and then at 30 °C. *Arrows* between the panels indicate cell additions. Details of the substrate and cell feeding can be found in Additional file 1: Table S1

The temperature in the multifeed SSCF was reduced from 35 to 30 °C after 24 h. At this point, the ethanol concentration had reached approximately 40–50 g L^−1^ (cf. Fig. 3). The reduction in temperature, together with cell feeding, led to increased viability and sustained fermentation capacity until the end of the process, with no accumulation of glucose (Fig. 6). In the 30 °C case, an increase in the fermentation rate was observed when a large amount of substrate was added at 120 h. In a similar process carried out at a constant temperature of 35 °C, glucose accumulated after the same substrate addition, indicating a decrease in fermentation, and a 26% lower ethanol yield on total sugars was measured at 168 h. The measured viable cells at the later stages were significantly lower in the process carried out isothermally at 35 °C, compared to the process with temperature reduction. The decrease in fermentation and accumulation of glucose started already between 96 and 120 h, indicating that the number of viable cells might be insufficient for taking up all glucose released via enzymatic hydrolysis (Fig. 6). Reducing the temperature likely decreases the rate of cellulose hydrolysis leading to lower glucose concentrations; however, very similar ethanol profiles were obtained for both cases before 120 h. This seems due to the enhanced xylose utilization observed in the process with a decrease in the temperature (Fig. 6). The glycerol formation followed the ethanol production (see Additional file 1: Figure S4, where also acetate, furfural, and HMF concentrations are shown).

In a previous study, Mutturi and Lidén instead applied a linear increase in temperatures ranging from 32 °C to 45 °C in SSF of pretreated spruce and Arundo [62]. When using Arundo, a higher ethanol titer was obtained in SSF with the temperature profile, than those in processes operated at a constant temperature of either 32 or 39 °C. This was likely due to the fact that, first, the increasing temperature would promote enzymatic hydrolysis compared with a constant temperature at 32 °C; and second, a gradual increase in temperature may give cells better chances to adapt than when using a constant high temperature from the beginning [62]. However, it is doubtful whether this strategy would work with more toxic substrates and at higher ethanol concentrations. In the same study, when pretreated spruce which contained higher amount of inhibitors was used, no difference in ethanol titers was observed between the processes with the temperature profile and with a constant temperature 39 °C (32.2 and 33.1 g L^−1^, respectively) [62]. These ethanol concentrations were considerably lower than the ones obtained herein, which may explain the different observations since they were below the level where we observed significant inhibition.

In conclusion, a temperature profile with a decrease in temperature after a certain period, combined with cell feeding, may offer a suitable process strategy for sustained viability and fermentation capacity at high ethanol titers.

### Adapting multifeed SSCF to KE-Flow and a new material batch increased ethanol production

As shown previously, the flocculating yeast strain KE-Flow performed similar to the parental KE6-12.A strain when it was propagated and used in fermentation processes that had been designed for KE6-12.A and material M1 (Fig. 3, Additional file 1: Figure S2; Table S2) [32]. As the material M2 had different compositions from that of M1 (Table 1), the media used in the propagation step and the feeding profile in the multifeed SSCF process were adjusted for the KE-Flow strain and material M2.

In the propagation, the objective was to maximize the use of the pretreatment liquor and minimize the use of molasses, while generating enough cells with consistent capacity for fermentation in SSCF. The medium containing 5% (v/v) molasses and 25% (v/v) pretreatment liquor was selected for the initial batch propagation, and the medium with 2.5% molasses and 80% pretreatment liquor was selected for fed-batch cultivation (for details see Additional file 1: Figure S5).

The objectives of re-designing the multifeed SSCF were to speed up the substrate feeding and to maximize the use of pretreatment liquor. Faster solids feeding, while avoiding mixing problems, is preferable because it prolongs the average time that substrates are available for hydrolysis. In a process with temperature reduction, a larger fraction of substrates would be loaded at the higher temperature, which may improve the overall hydrolysis.

The faster substrate feeding was developed by using the open loop approach described previously [32], with adjustments in the threshold values used to calculate when and how much substrate should be added. In brief, the rate of hydrolysis of the substrate was predicted using a kinetic model. When the predicted substrate conversion reached a lower threshold, 50% instead of the 60% that was used in the previous experiments, a feeding event was triggered. The amount of feed was calculated from the current concentration of WIS and the maximum concentration of WIS that could be adequately mixed in the reactor. For material M2, the lab reactors could mix medium containing up to 14% (w/w) WIS, while for material M1 the upper limit was 13% (w/w). The reason for the difference in the two material batches was probably that a sieve was used to homogenize the size of the solid clumps of M2 material at the demo plant. Starting feeds earlier and adding more substrates every time resulted in a faster substrate feeding profile (Additional file 1: Table S1). The new feeding was implemented successfully in laboratory-scale multifeed SSCF of material M2 without any mixing problems.

**Fig. 7:**
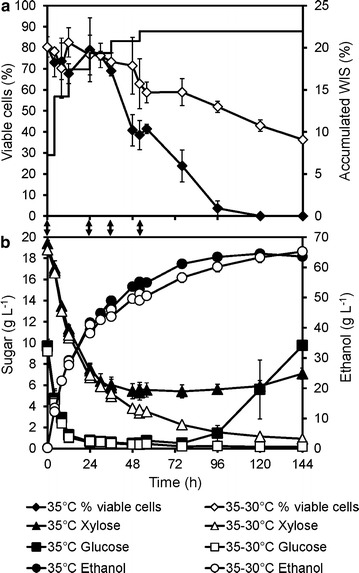
Adapted multifeed SSCF with faster feeding and maximum use of pretreatment liquor. **a** Cell viability (% of CFU/total cell counts) and accumulated WIS content (indicating substrate feeding); and **b** concentration of glucose, xylose, and ethanol in 22% (w/w) WIS multifeed SSCF of material M2, with the KE-Flow strain, at a constant temperature of 35 °C, or at 35 °C for 24 h, and then at 30 °C. *Arrows* between the *panels* indicate cell additions. Values are averages from duplicate experiments, and the *error bars* show the results of the individual experiments. Details of the substrate and cell feeding can be found in Additional file 1: Table S1

The use of pretreatment liquor in SSCF instead of water was also investigated together with the new feeding profile. The pretreatment liquor contained sugars, thus contributing to an increase in ethanol production in the early stage of the process (Fig. 7). Ethanol titers of greater than 60 g L^−1^ were obtained at 96 h, and 65 g L^−1^ was reached after 144 h of SSCF at 22% overall WIS, equivalent to 70% of the theoretical ethanol yield from the total sugar inputs. This represents a total process ethanol yield of 186 L/ton dry straw. The calculated yield at 120 h, which can be considered a more realistic process time, is given in Additional file 1: Table S2. The relatively low overall yields reflect the facts that some of the pretreated material is used to replace other sugars during cell propagation, and that both hydrolysis and fermentation were incomplete.

Decreasing the process temperature after 24 h improved the viability of the cells and the consumption of xylose and available glucose (Fig. 7). The measured glucose concentration was close to zero at the lower temperature. Between 24 and 72 h, less ethanol was produced than at the higher temperature. Therefore, less glucose must have been available for fermentation at the lower temperature, which means the hydrolysis rate was lower. The limited hydrolysis was also illustrated by the higher residual WIS and the proportion of glucose in the WIS at the end of the process, indicating a larger residual amount of unhydrolyzed cellulose at the lower temperature (Table 2). Glycerol was formed at higher concentrations at the lower temperature, which is compatible with improved growth and/or higher xylose conversion (Additional file 1: Figure S6). The high ethanol concentrations obtained until 96 h during isothermal operation at 35 °C illustrates the benefit of feeding cells during the process (Fig. 7). Further optimization is necessary for balancing the negative effect of a lower temperature on hydrolysis vs. the beneficial effects on ethanol tolerance, viability, and fermentative activity.

**Table 2:** Residual WIS (%, w/w) and sugar composition of the WIS [%, i.e., g (100 g WIS)^−1^] at 120 h of multifeed SSCF

Temperature of SSCF	WIS	Glucose	Xylose	Mannose	d-Galactose	l-Arabinose
35 °C	11.6 ± 0.8	11.9 ± 3.5	1.3 ± 0.2	1.1 ± 0.1	0.1 ± 0.0	0.2 ± 0.0
35 → 30 °C	13.0 ± 0.1	21.2 ± 0.6	1.7 ± 0.1	1.1 ± 0.0	0.0 ± 0.0	0.2 ± 0.0

The values given are the average of duplicate experiments ± the span to minimum and maximum values

### Scale-up: opportunities and challenges when using flocculating yeast and a temperature profile

To investigate the feasibility of using flocculating yeast in an industrial setting, the re-designed yeast propagation and multifeed SSCF processes (Additional file 1: Table S1) were carried out in 10 m^3^ reactors at the SP Biorefinery Demo Plant in Örnsköldsvik, Sweden. A new batch of material (M3) was produced. The furfural and xylose concentrations in the pretreatment liquor from this batch were higher than those in the M2 material used during the optimization of the laboratory-scale processes (the detailed compositions are given in Table 1).

When cultivated aerobically in the demonstration scale (10 m^3^) on the new material, the growth of the KE-Flow cells was similar to that in the laboratory during the batch phase and the fed-batch phase until the first cell withdrawal (Additional file 1: Figure S7). At the first harvest, cells were concentrated by sedimentation inside the propagation reactor, because agitation and aeration were stopped. A high cell density slurry (OD_600_ of ~200) was thus obtained by harvesting the cells from the bottom of the reactor. The cell slurry was used directly as inoculum for SSCF.

After the first harvest, the cells did not re-suspend properly upon restarting stirring and aeration. Cell growth was therefore insufficient for the planned subsequent cell feedings to the SSCF reactor (Additional file 1: Figure S7). Hence, the cell feeding that was planned at 12 h was omitted, and the corresponding amount of cells was instead added with the last two feedings (at 30 and 52 h). Using a sedimentation tank next to the propagation reactor, as was done at the laboratory scale, would have improved the process by reducing the disturbance of the propagation process.

**Fig. 8:**
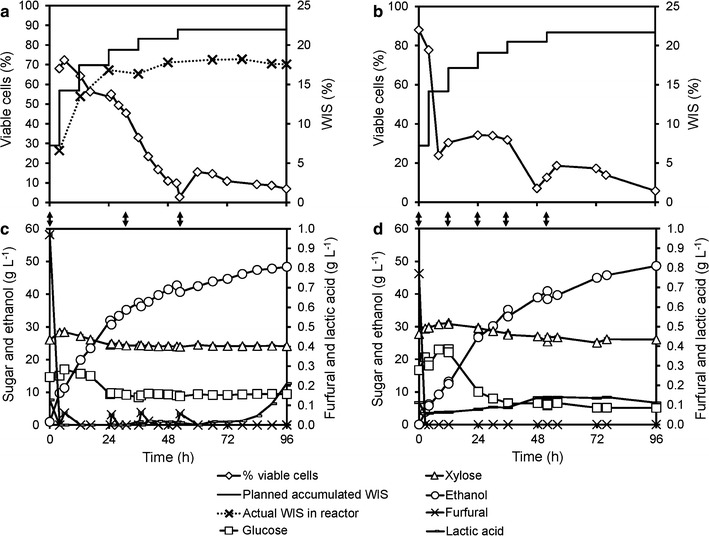
Multifeed SSCF of material M3 in 10 m^3^ and 30 L reactors with KE-Flow. SSCF was carried out at 35 °C for 48 h and then at 30 °C. **a** Cell viability (% of CFU/total cell counts), planned accumulation, and measured residual WIS (%, w/w) during the demonstration-scale experiment, and **b** the same experiment on an intermediate scale in a 30 L Techfors reactor; **c** concentration of sugars and fermentation products during the demo-scale experiment, and **d** during the reactor experiment. *Arrows* between the *panels* indicate cell additions

Since the hydrolysis rate was lower at the lower temperature of 30 °C (Table 2), further optimization of substrate feeding and temperature profile was required. The initial period of higher temperature was extended to 48 h in order to improve hydrolysis, and a final cell feeding was performed at 52 h. Actual cell dry weights added in three cell feedings were about 13, 1.3 and 3 kg, respectively. Before the last cell addition, the percentage of viable cells was 3%. After the temperature reduction, the decrease in viable cells was about 85% slower than during the 23 h (Fig. 8a) before the temperature reduction. Furthermore, the ethanol production rate appeared to be constant until the end of the process (Fig. 8c). In the end, the percentage of viable cells over total cells was approximately 8%. Of the cells added at the last feeding, approximately 45% remained viable, so the lower temperature clearly improved cell viability in the demonstration scale as well. However, the concentration of viable cells was much lower than in the laboratory-scale experiments at the corresponding time points, also after the decrease in temperature (Figs. 7a, 8a).

After 24 h of fermentation, the residual glucose concentration in the reactor leveled out at approximately 10 g L^−1^, and the xylose concentration at approximately 25 g L^−1^ (Fig. 8c). This indicates that fermentation was significantly slower than on the laboratory scale. The difference in performance was probably due to the problems encountered in handling the flocculating yeast and process control on the larger scale. It was found, for example, that the actual WIS content in the demo reactor was higher than planned, meaning that too much solid material or too little liquid had been added to the reactor. The resulting high viscosity led to reduced mixing, hydrolysis, and fermentation. Another reason may be differences in the toxicity of the material, as M3 contained significantly higher amounts of inhibitors than M2 (Table 1). The concentration of acetate in the fermentation broth increased to 4.6 g L^−1^ at 96 h, while the glycerol concentration was lower than those in previous experiments, reflecting both poor growth and low xylose consumption (Additional file 1: Figure S8).

Although many adjustments and compromises had to be made to operate multifeed SSCF at the demonstration plant, ethanol titers above 5% (w/w) were achieved. Toward the end of the process, small amounts of lactic acid were formed, and some bacterial colonies were found on the YPD plates used for CFU determination. However, since the lactate concentration was very low (<0.3 g L^−1^) and available glucose in the medium was high, contamination was not a major problem during these experiments.

The multifeed SSCF experiment could not be repeated on the demo scale due to limited resources, but was repeated on an intermediate scale in the laboratory using the same material. The demo process was scaled down to a 30 L Techfors reactor, i.e., roughly 10 times the scale of the 3.6 L Infors reactors used for process development. The fermentation results were consistent with those obtained from the demo plant experiment (Fig. 8; Additional file 1: Figure S8). Neither of them reached the high ethanol titers obtained during the laboratory-scale experiments using material M2 (Fig. 7). No issues with the mixing in the fermenter were observed during the experiment in the Techfors reactor. It could therefore be concluded that differences between the materials obtained after pretreatment were the main contributor to the different fermentation results.

Apart from demonstrating scalability of the process, the results from larger scales highlight the importance of consistency in the pretreatment process, in order to obtain material with similar hydrolyzability and inhibition characteristics. However, this is difficult to achieve due to variations in feedstocks and difficulties in operation on a large scale. Thus, flexibility in the SSCF process is necessary. The multifeed SSCF process is an example of an adaptable process. Adjustments can be made to the process based on the toxicity of the material by changing the amount of pretreatment liquor in the propagation step and in the SSCF, together with modifications of the feeding of cells and solids. To enable such adjustments in real time, rapid sampling methods and online measurements of sugars, inhibitors, and viable cells will be required to provide information on the status of the process, and to make feedback control of multifeed SSCF possible.

## Conclusions

Results from laboratory- and demonstration-scale experiments showed the feasibility of utilizing flocculating yeast cells as an efficient way to concentrate cells for use in multifeed SSCF. Ethanol inhibits fermentation at a concentration of 50 g L^−1^ in the presence of lignocellulosic inhibitors. Flocculation did not provide a solution to the ethanol inhibition in SSCF, but is an attractive strategy for facilitating cell harvesting and processing. The fermentation capacity of the cells can be maintained, and higher ethanol titers can be achieved by reducing the temperature in the SSCF reactor when the ethanol concentration reaches an inhibitory level. These changes, together with faster substrate feeding, and replacing water with pretreatment liquor, led to an ethanol titer of 65 g L^−1^ with maintained cell viability and almost complete utilization of the fermentable sugars. Optimal performance of multifeed SSCF requires adaptation of the process to each material used.

## Additional file

**Additional file 1: Figure S1.** Ethanol inhibition in high-gravity enzymatic hydrolysis with enzyme preparation Cellic Ctec 2. **Figure S2.** Shake flask SSCF with the non-flocculating strain KE6-12.A and the flocculating strain KE-Flow. **Figure S3.** Spotting assay of the B-Flow cells. **Figure S4.** Multi-feed SSCF at constant temperature or with temperature reduction. **Figure S5**. Investigation of medium compositions for batch and fed-batch propagation of KE-Flow with material M2. **Figure S6.** Adapted multi-feed SSCF with faster feeding and maximum use of pretreatment liquor. **Figure S7.** Comparison of cultivation of KE-Flow in 3.6 L laboratory reactors and in the demonstration plant. **Figure S8.** Multi-feed SSCF of material M3 in 10 m^3^ and 30 L reactors with KE-Flow. **Table S1.** SSCF feeding schemes for laboratory scale reactors. **Table S2.** Summary of results from multi-feed SSCF experiments using steam pre-treated wheat straw.
