# Supplementary material for: Sustaining fermentation in high-gravity ethanol production by feeding yeast to a temperature-profiled multifeed simultaneous saccharification and co-fermentation of wheat straw
Source: Biotechnol Biofuels. 2017 Sep 12;10:213. doi: 10.1186/s13068-017-0893-y (PMC5596858; doi:10.1186/s13068-017-0893-y)
Supplement: Supplementary file 1 — Additional file 1: Figure S1. Ethanol inhibition in high-gravity enzymatic hydrolysis with enzyme preparation Cellic Ctec 2. Figure S2. Shake flask SSCF with the non-flocculating strain KE6-12.A and the flocculating strain KE-Flow. Figure S3. Spotting assay of the B-Flow cells. Figure S4. Multi-feed SSCF at constant temperature or with temperature reduction. Figure S5. Investigation of medium compositions for batch and fed-batch propagation of KE-Flow with material M2. Figure S6. Adapted multi-feed SSCF with faster feeding and maximum use of pretreatment liquor. Figure S7. Comparison of cultivation of KE-Flow in 3.6 L laboratory reactors and in the demonstration plant. Figure S8. Multi-feed SSCF of material M3 in 10 m3 and 30 L reactors with KE-Flow. Table S1. SSCF feeding schemes for laboratory scale reactors. Table S2. Summary of results from multi-feed SSCF experiments using steam pre-treated wheat straw. [file 13068_2017_893_MOESM1_ESM.docx]

# Sustaining fermentation in high-gravity ethanol production by feeding yeast to a temperature-profiled multifeed simultaneous saccharification and co-fermentation of wheat straw

# Additional file

Johan O. Westman^1,2†^, Ruifei Wang^1†^, Vera Novy^1,3,4^, Carl Johan Franzén^1*^

^1^Division of Industrial Biotechnology, Department of Biology and Biological Engineering, Chalmers University of Technology, Gothenburg, Sweden

^2^Current address: Chr. Hansen A/S, Bøge Allé 10-12, DK-2970 Hørsholm, Denmark

^3^Institute of Biotechnology and Biochemical Engineering, Graz University of Technology, Graz, Austria

^4^Current address: Department of Chemical Engineering, Lund University, Lund, Sweden

^†^Contributed equally.

*Correspondence: [franzen@chalmers.se](mailto:franzen@chalmers.se).

**
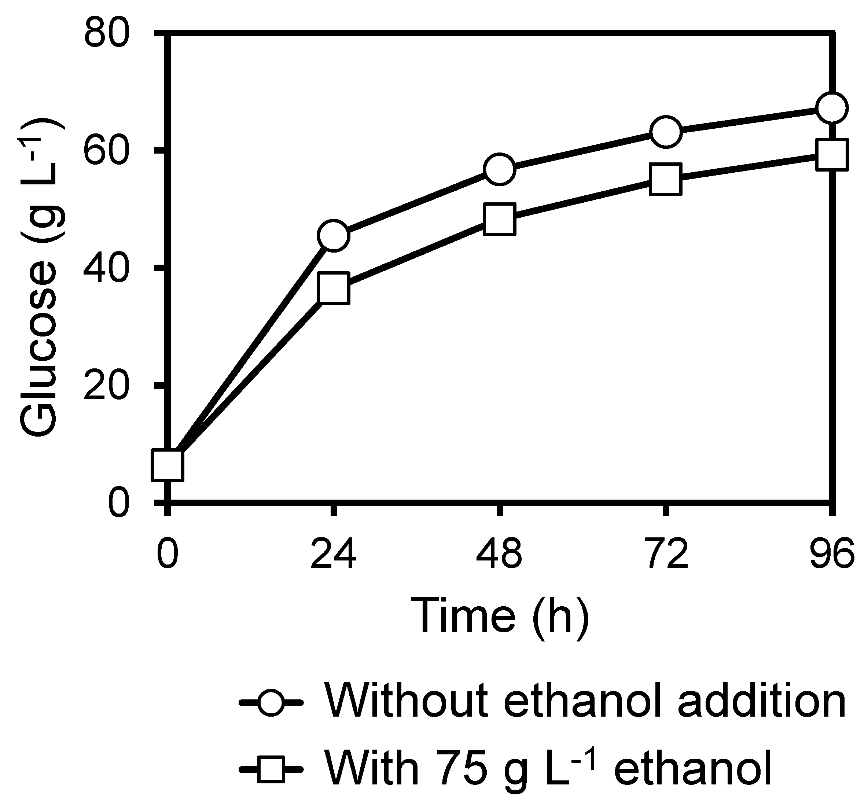
**

**Figure S1.** Ethanol inhibition in high-gravity enzymatic hydrolysis with enzyme preparation Cellic Ctec 2.

Shake flask hydrolysis of 20% (w/w) WIS of steam pretreated wheat straw (material M1), 10 FPU (g WIS)^-1^ enzyme dosage, at 35°C. Enzymatic hydrolysis was slightly inhibited by the initial addition of approximately 75 g L^-1^ ethanol.





**Figure S2.** Shake flask SSCF with the non-flocculating strain KE6-12.A and the flocculating strain KE-Flow.

Batch SSCF was conducted with 20% (w/w) WIS of material M1 and an enzyme dosage of 10 FPU (g WIS)^-1^. Pre-hydrolysis was performed for 2 h at 50°C before time 0. The temperature was then decreased to 35°C and cells were added to a concentration of 0.02 g cells (g WIS)^-1^. Results shown are average values of duplicate experiments, and the relative difference in ethanol concentration between duplicate experiments was below 5%.

**

**

**Figure S3.** Spotting assay of the B-Flow cells.

The B-Flow strain was created from IBB10B05 [1] using the same procedure as for the KE-Flow strain. YPD agar plates were supplemented with 50 g L^-1^ ethanol, or 50% (v/v) pretreatment liquor (from material M2), or both. The cells were harvested after 24 h of fed-batch propagation. The plates were incubated at 35°C or 30°C for 48 hours. Duplicate cell dilution and plating showed similar results.

**Figure S4.** Multifeed SSCF at constant temperature or with temperature reduction.

Concentrations of acetated, glycerol, furfural and HMF in 25% (w/w) WIS multifeed SSCF of material M2, with the KE-Flow strain, at a constant temperature of 35°C, or at 35°C for 24 h and then at 30°C. Details of the substrate and cell feeding can be found in Additional file: Table S1.

**
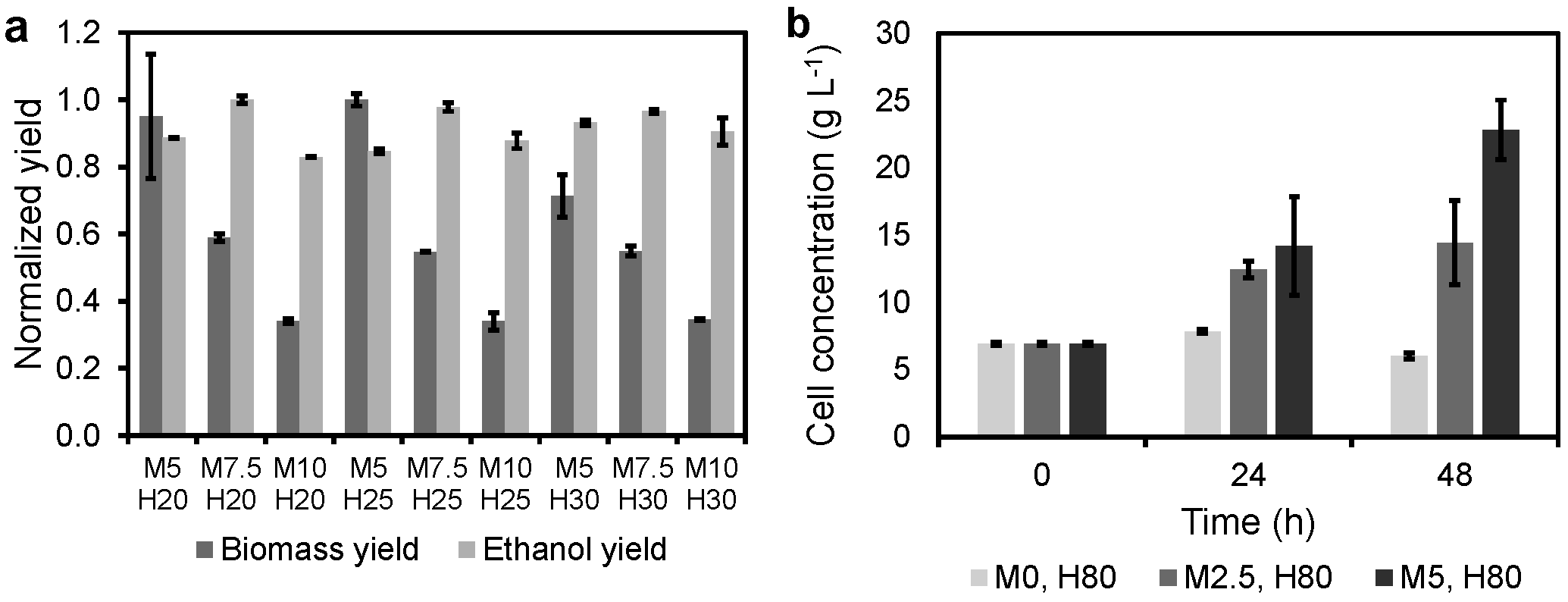
**

**Figure S5.** Investigation of medium compositions for batch and fed-batch propagation of KE-Flow with material M2.

(a) Biomass and ethanol yields during the 24 h shake flask batch cultivation in the indicated media, normalized to the highest calculated respective value of the yields. The M5-H25 medium was selected for batch because it led to the best combination of high biomass yield and high hydrolysate use, with complete utilization of the sugars. (b) Cell concentrations during fed-batch propagation at indicated time points, when indicated feeding media were used. The medium M2.5-H80 was selected since the aim was to minimize the molasses demand, while having stable cell concentration during the propagation and sufficient cells to be fed to the SSCF reactors. The fermentation capacity of the cells obtained from different media and different time points were found similar, in batch shake flask 20% (w/w) WIS SSCF, with 10 FPU (g WIS) ^-1^ enzyme dosage and 0.02 g dry weight (g WIS) ^-1^ cell dosage, at 35°C. M: Molasses, H: Hydrolysate (pretreatment liquor), numbers indicate volume percentage.

**Figure S6.** Adapted multifeed SSCF with faster feeding and maximum use of pretreatment liquor.

Concentrations of acetated, glycerol, furfural and HMF in 22% (w/w) WIS multifeed SSCF of material M2, with the KE-Flow strain, at a constant temperature of 35°C, or at 35°C for 24 h and then at 30°C. Values are averages from duplicate experiments, and the error bars show the results of the individual experiments. Details of the substrate and cell feeding can be found in Additional file: Table S1.





**Figure S7.** Comparison of cultivation of KE-Flow in 3.6 L laboratory reactors and in the demonstration plant.

The reduction in cell concentration (expressed as OD) during propagation illustrates the difficulties in cultivating flocculating yeast in the demonstration plant.

**Figure S8.** Multifeed SSCF of material M3 in 10 m^3^ and 30 L reactors with KE-Flow.

SSCF was carried out at 35°C for 48 h and then at 30°C. Concentrations of acetated, glycerol, furfural and HMF in the demonstration-scale experiment and on an intermediate scale in a 30 L Techfors reactor.

**Table S1.** SSCF feeding schemes for laboratory scale reactors

| **Feeding profile for multifeed SSCF ^a^** | | | | | | | | | | |
| --- | --- | --- | --- | --- | --- | --- | --- | --- | --- | --- |
| **Time (h)** | **Solid (g)** | **H_2_O (g)** | **Acc. WIS (%)** | **Enzyme (FPU)** | **Enzyme (ml)** | **Cells (g)** | **Cells (ml)** | **Total weight (g)** | **WIS (g)** | **Total liquid (g)** |
| **0** | 98 | 314 | 7.0 | 2783 | 18.6 ^c^ | 2.85 | 80 ^d^ | 592 | 41.5 | 551 |
| **4** | 136 | 14 | 13.3 |  |  |  |  | 742 | 57.3 | 643 |
| **12** | 104 | 11 | 16.7 |  |  |  |  | 857 | 43.9 | 715 |
| **24** | 90 |  | 18.7 |  |  | 0.76 | 19.6 ^d^ | 967 | 38.2 | 786 |
| **48** | 81 |  | 20.2 |  |  | **0.69** | 18.7 ^d^ | 1067 | 34.4 | 852 |
| **72** | 77 |  | 21.3 |  |  | **0.65** | 18.2 ^d^ | 1162 | 32.5 | 914 |
| **96** | 72 |  | 22.2 |  |  | 0.61 | 17.7 ^d^ | 1252 | 30.6 | 974 |
| **120 ^b^** | 201 ^b^ |  | 25 ^b^ |  |  |  |  | 1453 ^b^ | 84.9 ^b^ | 1090 ^b^ |
| **Sum** | 659 (860 ^b^) | 340 |  |  | 100 ^c^ | 5.57 | 154 ^d^ | 1252 (1453^b^) | 278 (363 ^b^) | 974  (1090 ^b^) |
| **Faster feeding profile for multifeed SSCF ^e^** | | | | | | | | | | |
| **Time (h)** | **Solid (g)** | **Liquor (g)** | **Acc. WIS (%)** | **Enzyme (FPU)** | **Enzyme (ml)** | **Cells (g)** | **Cells (ml)** | **Total weight (g)** | **WIS (g)** | **Total liquid (g)** |
| **0** | 100 | 354 | 7.2 | 2345 | 15.7 ^c^ | 2.88 | 30 ^f^ | 584 | 42.3 | 541 |
| **4** | 145 |  | 14.2 |  |  |  |  | 729 | 61.3 | 625 |
| **12** | 95 |  | 17.4 |  |  |  |  | 824 | 40.1 | 680 |
| **24** | 80 |  | 19.4 |  |  | 0.68 | 12 ^f^ | 916 | 33.8 | 738 |
| **36** | 70 |  | 20.8 |  |  | 0.59 | 10 ^f^ | 996 | 29.6 | 789 |
| **52** | 65 |  | 22.0 |  |  | 0.55 | 8 ^f^ | 1069 | 27.5 | 834 |
| **Sum** | 555 | 354 |  |  | 100 ^c^ | 4.69 | 60 ^f^ | 1069 | 234.5 | 834 |

^a^ Feeding of solid substrate to reach the instantaneous WIS content of 13 % (w/w) was carried out when 60 % of the cellulose was hydrolyzed, based on simulation results according to **[2]**.

^b^ With extra solids feeding to 25% WIS

^c^ Diluted to 100 ml with deionized water or pre-treatment liquor.

^d^ Resuspension volume of harvested and centrifuged cell culture with pre-treatment liquor.

^e^ Feeding of solid substrate to reach the instantaneous WIS content of 14 % (w/w) was carried out when 50 % of the cellulose was hydrolyzed, based on simulation results according to **[2]**.

^f^ Cell suspension volume of the sedimented cells.

**Table S2.** Summary of results from multifeed SSCF experiments using steam pre-treated wheat straw.

| **Strain: material** | **Feedings** | **Temp profile^a^** | **Final WIS ^b^** | **Y_E_ ^c^**  **96 h** | **Y_E_**  **120 h** | **Y’_E_ ^e^ 120h** | **Glucose**  **96 h** | **Glucose**  **120 h** | **H2O:Liquor (g WIS)^-1^ ^f^** | **EtOH^g^ L/TDM** |
| --- | --- | --- | --- | --- | --- | --- | --- | --- | --- | --- |
| **KE6-12.A:M1** | As [2] | No | 22.2 | 65.6±0.9 | 60.7±0.5 | n.a. | 4.7±1.0 | 9.9±0.5 | 1.7:0 | 173 |
| **KE-Flow:M1** | As [2] | No | 22.2 | 60.0±1.9 | 54.4±1.8 | n.a | 12.7±4.4 | 21.5±3.3 | 1.7:0 | 155 |
| **KE-Flow:M2** | As [2] | No | 22.2 | 71.4 | 68.5^d^ | n.a | 0.36 | 2.87 | 2:0 | 160 |
| **KE-Flow:M2** | As [2] | 24h | 22.2 | 70.1 | 70.9^d^ | n.a | 0.31 | 0.26 | 2:0 | 166 |
| **KE-Flow:M2** | New | No | 22.0 | 70.0±0.4 | 71.2±1.3 | 87.8±2.2 | 1.5±0.7 | 5.6±2.8 | 0:1.5 | 183 |
| **KE-Flow:M2** | New | 24 h | 22.0 | 66.4±1.9 | 69.9±1.9 | 91.2±1.0 | 0.2±0.0 | 0.2±0.1 | 0:1.5 | 179 |
| **B-Flow:M2** | New | No | 22.0 | 67.4±2.0 | 69.2±0.5 | n.a | 2.0±1.3 | 5.4±4.2 | 0:1.5 | 177 |
| **B-Flow:M2** | New | 24 h | 22.0 | 60.7±2.4 | 64.6±2.3 | n.a. | 0.6±0.1 | 0.5±0.1 | 0:1.5 | 166 |
| **KE-Flow:M3 ^h^** | New | 48 h | 21.7 | 53.3 | 55.9 | n.a | 5.1 | 6.5 | 0.2:1.5 | 152 |
| **KE-Flow:M3 ^i^** | New | 48 h | 21.8 | 55.1 | 56.7 | 68.2 | 9.4 | 9.2 | 0.2:1.8 | 151 |

Data show are mean ± the span to minimum and maximum values of biological duplicate experiments.

^a^ *No* indicates that SSCF was carried out at 35°C throughout. Otherwise multifeed SSCF was operated at 35°C until the indicated time, then the process temperature was kept at 30°C.

^b^ Planned overall WIS (%, w/w) after all feedings.

^c^ Y_E_ stands for % of the theoretical overall ethanol yield on all sugar inputs to the bioreactor. (${Y_{E}=Concentration}_{EtOH}\times V_{liquid}/0.51/(Sugar_{WIS}+Sugar_{liquor}+Sugar_{Enzyme})$)

^d^ An extra substrate addition was conducted at 120h, the ethanol yield at 168h for SSF run at 35°C was 52.9%, while under 30°C the ethanol yield was 67.9% , clearly indicating the benefit of applying a temperature profile for maintaining the fermentation capacity.

^e^ Y’_E_ stands for % of the theoretical ethanol yield on consumed sugars. The consumed sugars were calculated by subtracting remaining sugars in the liquid phase and in the residual WIS in the SSCF broth from the total sugar input.

^f^ The volume ratio of water and pretreament liquor used in multifeed SSCF.

^g^ The EtOH L/Ton Dry straw was calculated by dividing the produced ethanol at 120 h by original straw dry mass calculated from the loaded total WIS in such experiments.

^h^ Multifeed SSCF carried out in 30 L laboratory reactor.

^i^ Multifeed SSCF carried out in 10 m^3^ reactor at the Demo Plant.

References

1. Klimacek M, Kirl E, Krahulec S, Longus K, Novy V, Nidetzky B. Stepwise metabolic adaption from pure metabolization to balanced anaerobic growth on xylose explored for recombinant *Saccharomyces cerevisiae*. Microb Cell Fact. 2014;13:37.

2. Wang R, Unrean P, Franzén CJ. Model-based optimization and scale-up of multi-feed simultaneous saccharification and co-fermentation of steam pre-treated lignocellulose enables high gravity ethanol production. Biotechnol Biofuels. 2016;9:88.
